# Ultra-fast-track extubation is associated with reduced EEG abnormalities and improved early outcomes after pediatric cardiac surgery: a propensity score-matched cohort study

**DOI:** 10.1186/s13019-026-04153-2

**Published:** 2026-05-05

**Authors:** Xiaowei Li, Rouyi Lin, Na Du, Jinqing Feng, Na Zhou, Shuyao Ning, Xinxin Chen, Li Ma, Mingjie Zhang, Huaizhen Wang, Jia Li

**Affiliations:** 1 Guangdong Provincial Key Laboratory of Research in Structural Birth Defect Disease, Women and Children’s Medical Center Affiliated to Guangzhou Medical University, Guangdong, China; 2 Cardiac Intensive Care Unit, Women and Children’s Medical Center Affiliated to Guangzhou Medical University, Guangdong, China; 3 Clinical Physiology Laboratory, Institute of Pediatrics, Women and Children’s Medical Center Affiliated to Guangzhou Medical University, No.9 Jinsui Road, Tianhe District, Guangzhou, Guangdong Province China; 4 Department of Electroneurophysiology, Women and Children’s Medical Center Affiliated to Guangzhou Medical University, Guangdong, China; 5 Department of Radiology, Women and Children’s Medical Center Affiliated to Guangzhou Medical University, Guangdong, China; 6 Anesthesiology department, Women and Children’s Medical Center Affiliated to Guangzhou Medical University, Guangdong, China

**Keywords:** Ultra-fast-track extubation, Electroencephalography, EEG background and abnormalities, Pediatric cardiac surgery

## Abstract

Ultra-fast-track extubation (UF) improves postoperative recovery in cardiac surgery, but its cerebral effects remain unclear. This study compared UF and conventional extubation (CE) in children with congenital heart disease (CHD) after cardiopulmonary bypass (CPB), focusing on electroencephalographic (EEG) abnormalities during the initial 48 postoperative hours. Of 352 CHD patients undergoing CPB, 57 UF and 295 CE cases were propensity score-matched (PSM) (1:2), yielding 55 PSM-UF and 89 PSM-CE subjects. Intra/postoperative EEGs were analyzed for background abnormalities (sleep-wake cycle) and epileptiform discharges (seizures, spikes/sharp waves). Clinical parameters including STS-EACTS mortality risk and CPB duration were balanced. The PSM-UF group demonstrated milder background abnormalities (*P* = 0.02) and lower incidence of unresolved abnormalities at 48 h (7% vs. 24%, *P* = 0.009). Epileptiform activity was significantly reduced (0% vs. 11% seizures, *P* = 0.007; *P* = 0.008 for spikes/sharp waves). UF patients showed superior cerebral oxygen saturation (ScO_2_, *P* < 0.0001), reduced vasopressor requirements (*P* < 0.0001), and shorter hospital stays (2.0 ± 1.4 vs. 6.0 ± 5.6 days, *P* < 0.0001) with comparable CICU stay reductions (9.6 ± 4.1 vs. 13.3 ± 8.5 days, *P* = 0.002). UF following pediatric cardiac surgery correlates with attenuated EEG abnormalities and enhanced early recovery, supporting its neuroprotective benefits in CHD patients.

## Introduction

With the improvement of cardiopulmonary bypass (CPB) surgery techniques and perioperative management for patients with congenital heart disease (CHD), ultra-fast-track extubation (UF) is used more frequently, particularly in patients with relatively simple CHD needing cardiac surgeries. Evidence suggests that it enhances postoperative recovery and hemodynamic stability, resulting in shorter hospital stays and improved overall organ function [1–3]. Perioperative brain injury and long-term neurodevelopmental impairment are serious complications in patients undergoing CPB, particularly in infants [2, 4]. EEG is frequently used to identify abnormalities in the nervous system, particularly in cases of clinically silent brain lesions [5, 6]. In a previous study, we comprehensively assessed postoperative EEG background (including sleep-wake cycling, SWC) and discharge abnormalities (such as seizures and spikes/sharp waves). We found that these abnormalities were significantly linked to adverse early postoperative outcomes [7]. This study aimed to examine the differences in postoperative EEG abnormalities and early clinical outcomes in children between the UF and CE groups. We focused on the initial 48 h after cardiac surgery using the propensity score matching method.

## Materials and methods

### Patient population

**Fig. 1:**
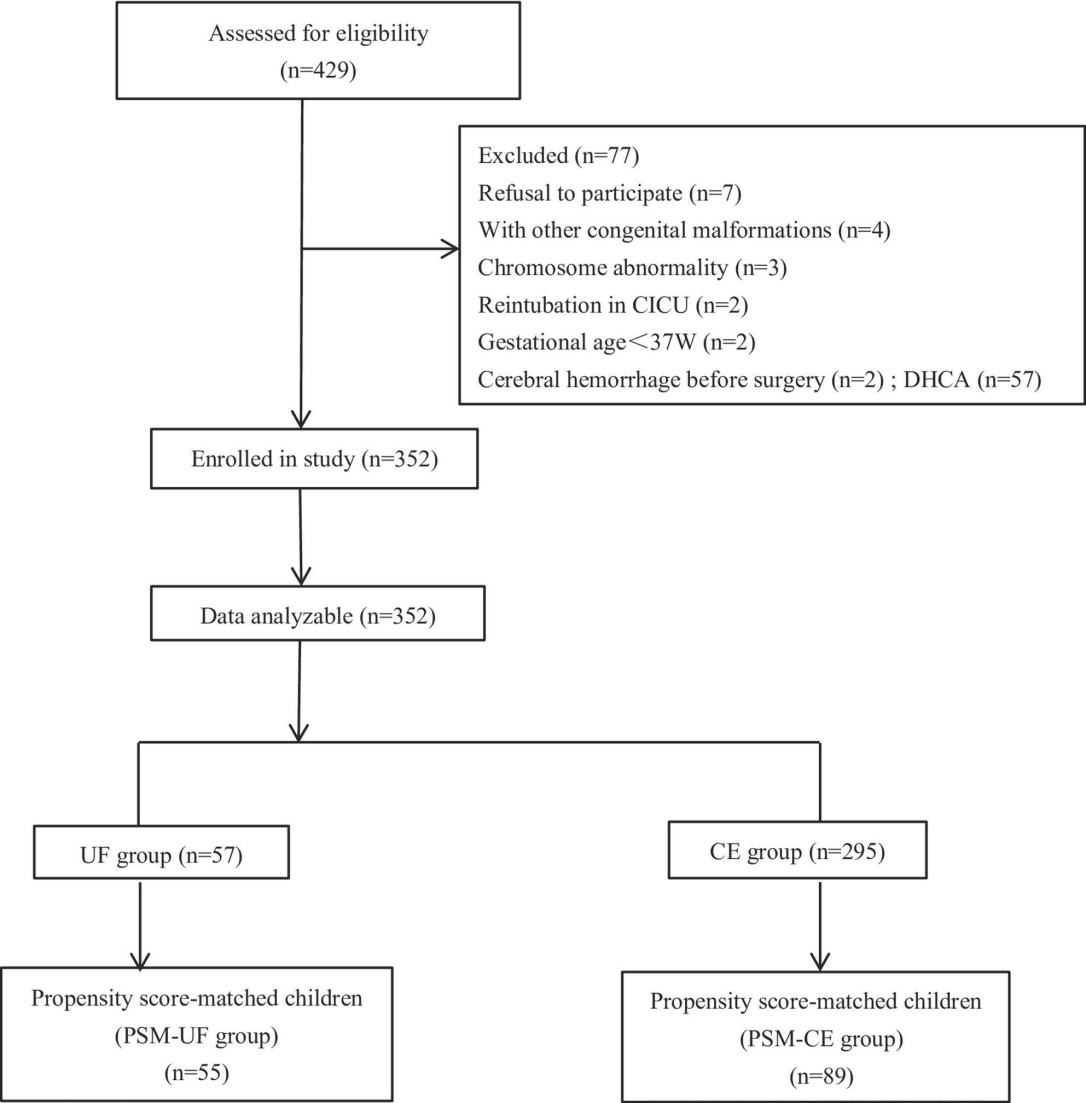
Study flow diagram displaying patient inclusion and exclusion criteria

A total of 352 patients who underwent CPB were prospectively enrolled, and EEG monitoring was performed from January 2020 to December 2023. All patients had uneventful recovery without major complications (cardiac arrest or the use of ECMO). Patients with more complex CHD were selected from the daily operation lists that were categorized by the STS-EACTS mortality guidelines [8]. Patients were excluded for the following reasons: (1) gestational age < 37 weeks at birth; (2) genetic syndromes or associated with other congenital anomalies; (3) reintubation in the operating room or CICU; (4) Preoperative cerebral hemorrhage; (5) Use of deep hypothermic circulatory arrest (DHCA) (see Fig. 1).

### Intraoperative procedures

Upon entering the operating room, a preoperative IV dose of penehyclidine hydrochloride was administered at 0.01mg$\cdot$kg.^− 1^. Anesthesia was induced by administering propofol (2 ~ 3mg$\cdot$kg.^− 1^), cisatracurium (0.2 ~ 0.3mg$\cdot$kg.^− 1^), and sufentanil (0.5 ~ 1mcg$\cdot$kg.^− 1^), followed by tracheal intubation and monitoring of arterial pressure, central venous pressure, end-tidal CO_2_, and urine volume. Sevoflurane was continuously administered during the procedure until the aortic cross-clamp was released.

Standard CPB procedures were performed. The priming solution consisted of packed red blood cells mixed with Lactated Ringer’s solution. Cold crystalloid cardioplegia (Custodiol HTK-Solution 50ml$\cdot$kg.^− 1^) was delivered after aortic cross-clamping. Nonpulsatile and low-flow CPB (100∼200ml$\cdot$kg.^− 1^min.$\cdot$^− 1^ was performed with 28∼32 °C hypothermia) was maintained during surgery. The mean perfusion pressure was adjusted to a range of 30 to 50mmHg with the use of sevoflurane and phenylephrine. The CPB was weaned after modified ultrafiltration, once the nasopharyngeal temperature reached 36 °C and hemodynamic stability was established. At the end of CPB, dopamine was continuously administered at a rate of 5 ~ 10mcg$\cdot$kg.^− 1^min.$\cdot$^− 1^, along with milrinone at a rate of 0.3 ~ 0.75mcg$\cdot$kg.^− 1^min.$\cdot$^− 1^, to ensure stable hemodynamics. If needed, epinephrine (0.05 ~ 0.2mcg$\cdot$kg.^− 1^min.$\cdot$^− 1^) was administered to maintain adequate blood pressure. When venous blood from the superior and inferior caval veins reflows into the right atrium, mechanical ventilation was restarted. There was no difference in anesthesia induction and maintenance between two groups.

Assessment was conducted comprehensively in four aspects (surgical, respiratory, circulatory and intra-environmental) throughout the procedure in all patients in order to evaluate whether extubation in the operating room. (1) STS-EACTS Mortality Categories < 5 (There are no Category 6 patients undergoing CPB in our center); (2) TEE evaluation of the operation results was basically satisfactory; (3) The results of arterial blood gas analysis (including SaO_2_, PaO_2_, PaCO_2_ and serum lactate, ) reached normal value by the end of CPB; (4) Ventilator parameters (including tidal volume, respiratory rate) reached normal value at extubation; (5) Lung compliance reached normal value at extubation; (6) TEE evaluation showed that myocardial contractility was weakened or normal and myocardial volume was slightly lower or normal; (7) The ratio of the systemic pulmonary circulation pressure was < 0.75; (8) The vascular active drug score was < 20; (9) Hb (g.dL^− 1^) was higher than the critical value; (10) Lactate (mmol.L^− 1^) was < 3; 11. Urine output (ml.kg^− 1^.h^− 1^) was > 0.5; 12. Central body temperature was > 35 °C.

After closing the sternum, the endotracheal tube was removed if the patient was breathing spontaneously and met the following criteria: tidal volume greater than 5 ml/kg, respiratory rate less than 40 breaths per minute, and arterial oxygen saturation (SpO_2_) was at least 95% with an FiO_2_ of 0.6 or lower. The patient then received high-flow nasal cannula oxygenation in the CICU. If the patient did not meet the above criteria, they were returned to the CICU with endotracheal tube and mechanical ventilation.

### Postoperative sedation management in CICU

After surgery, both groups received Patient-Controlled Analgesia Pump, involving a regimen of dexmedetomidine (0.3mcg$\cdot$kg-1.h-1) and sufentanil (0.03mcg.kg-1.h-1). Additionally, patients received intermittent IV midazolam (0.1 ~ 0.3 mg.$\cdot$kg-1 per dose) as needed. The vasoactive drugs used to maintain arterial blood pressure included dopamine (5 ~ 10mcg.kg-1.min-1), milrinone (5 ~ 10mcg.kg-1.min-1), and epinephrine (0.05 ~ 0.2mcg.kg-1.min-1), with target systolic pressures of 60 to 90mmHg for neonates and 90 to 105mmHg for children..

### EEG

Continuous Video-EEG was recorded using Nicolet monitor (CareFusion, Middleton, Wisconsin, USA) during operation and within 48 h after cardiac surgery. The recording of electrical activity was collected from scalp electrodes positioned in the FP1, C3, T3, O1, Cz, FP2, C4, T4 and O_2_ positions according to the international 10–20 system. The degree of EEG background was categorized and graded from 0 to 4 as: 0) normal; (1) slow-disorganized; (2) discontinuous; (3) burst-suppression; (4) attenuated-featureless [9]. Abnormal SWC was defined as the lack of SWC in neonates; and as the lack of stage 2 transients (K-complexes and spindles) in older children [9, 10]. EEGs shown as transient (1–2 h) immediately after any sedative administration composed of slow waves superimposed with fast rhythms were excluded from the analysis. The isoelectric state was defined as no brain electrical activity for ≥3 min, amplitude < 10µV [11]. Electrographic seizures was defined as epileptiform discharges averaging > 2.5 Hz for ≥ 10 s, and status epilepticus as continuous seizures ≥ 10 min or for a total duration of ≥ 20% of any 60-minute period of recording [12]. The origin of seizures indicates the region of the initial seizure onset, and the spread region indicates the region that seizures spread to. Spikes/sharp waves were defined as high amplitude (≥ 2.5times of background voltage) and short duration (< 200ms) [13]. All EEGs were analyzed in 3-hour periods by the qualified technicians (RL and SN) independently and finalized by SN. EEG was recorded during operation only in some patients.

### Cerebral oxygen saturation (ScO_2_)

Near-infrared spectroscopy 9 (INVOS 5100 C, Medtronic & Covidien, Troy, MI, USA) was used to continuously monitor ScO_2_ which is the equilibrium of oxyhaemoglobin and deoxyhaemoglobin in a mixture of veins, arteries and capillaries in the underlying tissue and reflects a regional state of oxygenation. The sensors were placed on the children’s forehead below the hairline to the right and left of the midline. Averaged bilateral ScO_2_ was used and recorded every 3 h.

### Middle cerebral artery (MCA) velocity

The middle cerebral artery velocity (including systolic velocity, diastole velocity, mean velocity) was measured with transcranial Doppler (TCD) with a 2 MHz pulse-wave ultrasound transducer, which was fixed above the zygomaticarch (Multi-DopT; DWL Elektronische Systeme GmbH, Sipplingen, Germany) and interrogated the portion of the middle cerebral artery near its junction with the anteriorcerebral artery. Mean, systolic and diastolic MAC velocities (mMAC, sMAC, dMAC) were recorded on postoperative day 0, day 1, and day 2.

### Cerebral MRI

MRI scans were performed on a 3T Magentom Prisma scanner (Siemens, Munich, Germany) including standard T1, T2, diffusion-weighted imaging, and diffusion-tensor imaging at the median 9 (3–37) days after surgery. Brain injuries included white matter injury, stroke, and hemorrhage and were graded as mild, moderate, and severe.

### Clinical data

Demographic data, STS-EACTS Mortality Category [14], duration of CPB, CICU and hospital stay (see Table 1) were collected. Clinical measurements, including arterial blood gases, blood pressure, heart rate and doses of vasoactive agents, were recorded every three hours.

### Statistical analysis

Data were described as mean (SD), median (25th, 75th percentile) or frequency (%) when appropriate. Propensity score matching was conducted in a 1:2 fashion where possible with replacement as a sensitivity analysis. A calliper value of 0.2 was used, equal to 0.2 of the SD of the logit of the propensity score. The model selection strategy included clinically relevant confounders or covariates including age, sex, STS-EACTS Mortality Category and CPB time. Unpaired t test was used for comparison of the means between the two groups. The Mann-Whitney U-test was used for non-normal distribution variables and the Chi-squared test for categorical variables. Mixed linear regression for repeated measures was used to analyze the overall trends (*P*_*time*_) of variables and compare their difference between groups (*P*_*group*_). The interaction of time and group (*P*_*group×time*_) indicates the difference in trends between the groups. When a variable had a non-linear relationship with time, polynomial transformation was used for the best fit for time; the parameter estimate and *P*-value of time (parameter estimate_time_ and *P*_*time*_) indicated an earlier trend, and those for time^2^ (parameter estimate_time_^2^ and *P*_time_^2^ ) indicated a later trend. A *P*-value < 0.05 was considered statistically significant. (SAS 9.4, Cary, NC, the USA).

## Result

### Comparison between UF and CE groups before propensity score matching

**Fig. 2:**
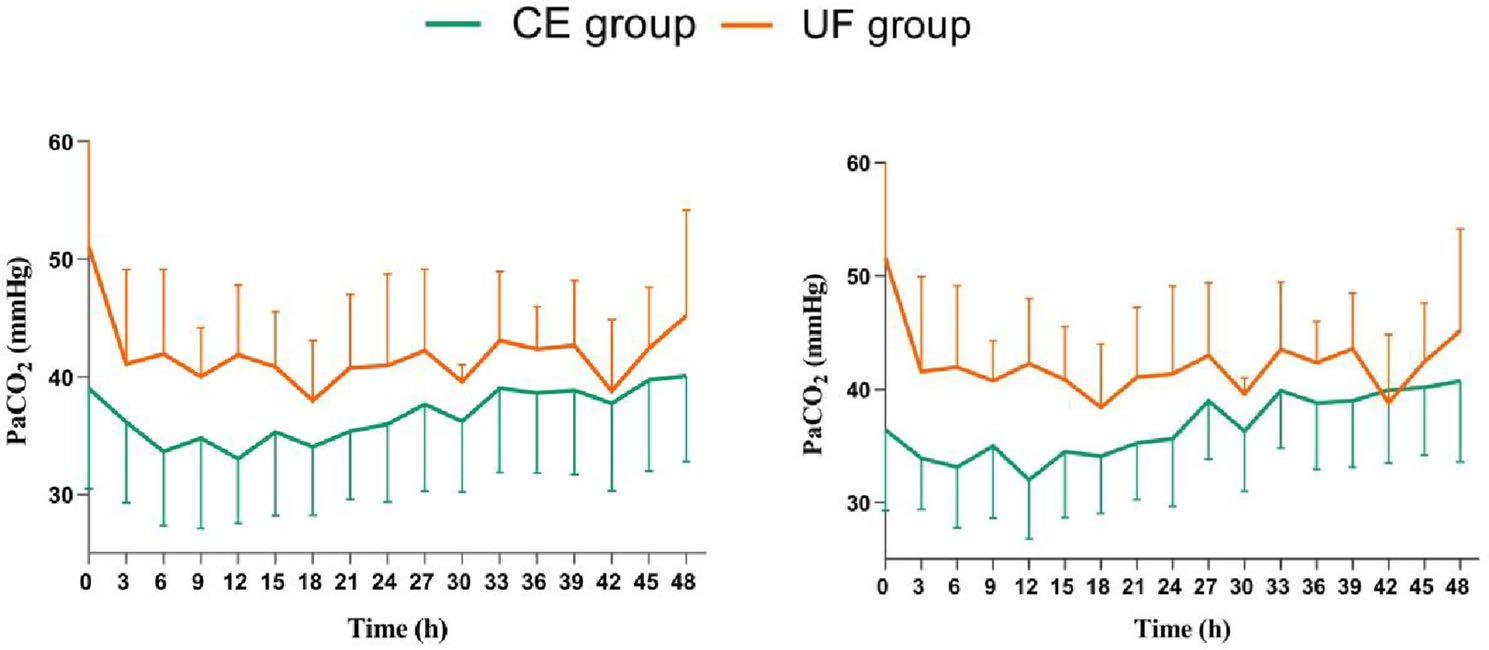
**A** The trend of the PaCO2 before propensity score matching. **B** The trend of the PaCO2 after propensity score matching

Before propensity score matching, there were statistically significant differences in age (*P* < 0.0001), body surface area (BSA) (*P* < 0.0001), CPB time (*P* = 0.001), and STS-EACTS Mortality Category (*P* < 0.0001) between the CE (*n* = 295) and UF (*n* = 57) groups. No seizures occurred in either group during the operation. After operation, the incidences of the lack of return to normal background by 48 h (*P* < 0.001) and seizures (*P* = 0.01) were significantly less in UF group. The incidence of the lack of return to normal SWC by 48 h was not significantly different between the two groups (*P* = 0.40) (see Table 1). The degree of background abnormalities and number of spike/sharp waves significantly decreased over 48 h in both groups (*P*_*time*_ < 0.0001 for both), and were significantly less in UF group compared to CE group (*P*_*group*_ = 0.002 for both) (see Table 2). ScO_2_ increased over 48 h in both groups (*P*_*time*_ < 0.0001), and was higher in UF group (*P*_*group*_ < 0.0001). The MAC velocities, including mMAC, sMAC, and dMAC, increased significantly in both groups over 48 h (*Ps* < 0.0001), and were significantly higher in UF group (*Ps* < 0.0001) as shown in Table 2. SaO_2_ increased over 48 h in both groups (*P*_*time*_ = 0.0002), but the level was not different between the two groups (*P*_*group*_ = 0.14). PaO_2_ decreased over 48 h in both groups (*P*_*time*_ = 0.0003), and it was higher in UF group (*P*_*group*_ = 0.003). PaCO_2_ in UF group was initially high (51mmHg SD) and significantly related to time after polynomial transformation over 48 h, with an early decrease in the first 12 h (parameter estimate_time_ = -0.44, *P*_*time*_ = 0.01) and followed by a gradual increase thereafter (parameter estimate_time_^2^ = 0.009, *P*_time_^2^ = 0.01). The overall level of PaCO_2_ over 48 h was significantly higher in UF group compared to CE group (parameter estimate = 6.43, *P* < 0.0001) (see Table 2). The trend of PaCO_2_ in UF and CE groups was showed in Fig. 2A and B. SBP and DBP decreased over time in both groups (*P*_*time*_ < 0.0001 for both), and SBP was significantly higher in UF group (*P*_*group*_ = 0.03) but not DBP (*P*_*group*_ = 0.37). Heart rate significantly increased over time in both groups (*P*_*time*_ = 0.04), but was lower in UF group (*P*_*group*_ = 0.0005) (see Table 2). Additionally, the dose of milrinone, epinephrine, and dopamine significantly decreased in both groups over 48 h (*Ps* < 0.0001), and were significantly lower in UF group (*Ps* < 0.0001). Lactate levels decreased in both groups during the 48 h following surgery (*P*_*time*_ < 0.0001). However, in contrast to the vasoactive drug dosages, there was no significant difference between the two groups (*P* = 0.002) (see Table 2). Both CICU and hospital stay durations were significantly shorter (*Ps* < 0.0001) in UF group. The brain injuries detected by MRI before discharge were not statistically different between the two groups (*P* = 0.74) (see Table 1).

**Table 1:** Clinical characteristics in UF and CE groups before propensity score matching

Variable	CE(*n* = 29)	UF(*n* = 5)	*P*-value
Sex, n (%)			0.93
Male	169(57)	33(58)	
Female	126(43)	24(42)	
Age (day)	124 ± 113	212 ± 101	< 0.001
Weight(kg)	4.95 ± 1.69	6.64 ± 1.9	< 0.001
BSA (m^2^)	0.28 ± 0.07	0.35 ± 0.5	< 0.0001
CPB time (min)	105 ± 58	80 ± 31	0.001
STS-EACTS Mortality Category, n (%)			< 0.0001
1	84(29)	35(61)	
2	117(40)	16(28)	
3	45(15)	3(5)	
4	49(17)	3(5)	
EEG abnormalities (*n* = 352)			
Seizures, n (%)	26(9)	0(0)	0.01
Lack of return to normal background by the 48th h, n (%)	86(29)	4(7)	< 0.0001
Lack of return to normal SWC by the 48th h, n (%)	23(8)	2(4)	0.40
Brain injury degree on MRI (*n* = 204)			0.74
Normal, n (%)	77(41)	8(57)	
Mild, n (%)	99(52)	6(43)	
Moderate, n (%)	6(3)	0(0)	
Severe, n (%)	8(4)	0(0)	
Length of CICU stay (day)	7.4 ± 12.0	2 ± 1.4	< 0.0001
Length of hospital stay (day)	15.5 ± 13.9	9.5 ± 4.1	< 0.0001

*BSA* body surface area; *CPB* cardiopulmonary bypass; *STS-EACTS* Society of Thoracic Surgeons-European Association for Cardio-Thoracic Surgery; *CICU* cardiac intensive care unit; *SWC* sleep-wake cycling

**Table 2:** Comparison of the continuous variables of EEG, hemodynamics, inotropes and ScO_2_ during the first 48 h after CPB between UF and CE groups before propensity score matching

Variables	Time		Group	
	Parameter estimate	*P*-value	Parameter estimate	*P*-value
Degree of EEG background abnormalities	-0.005	< 0.0001	-0.32	0.002
Spikes/sharp waves (times.min^− 1^)	-0.38	< 0.0001	-24.57	0.0002
Heart rate (times.min^− 1^)	0.03	0.04	-10.91	0.0005
SBP (mmHg)	-0.06	< 0.0001	3.39	0.0276
DBP (mmHg)	-0.11	< 0.0001	0.86	0.3729
VmMCA (cm.second^− 1^)	0.64	< 0.0001	12.24	< 0.0001
VsMCA (cm.second^− 1^)	0.84	< 0.0001	16.55	< 0.0001
VdMCA (cm.second^− 1^)	0.46	< 0.0001	8.54	< 0.0001
ScO_2_ (%)	0.38	< 0.0001	8.74	< 0.0001
PaO_2_ (mmHg)	-0.22	0.0003	17.71	0.0034
SaO_2_ (%)	0.02	0.0002	1.16	0.1432
PaCO_2_ (mmHg)	-0.44 0.009	0.01 0.01	6.43	< 0.0001
Milrinone (mcg.kg^− 1^.min^− 1^)	-0.002	< 0.0001	-0.20	< 0.0001
Epinephrine (mcg.kg^− 1^.min^− 1^)	-0.0006	< 0.0001	-0.04	< 0.0001
Dopamine (mcg.kg^− 1^.min^− 1^)	-0.03	< 0.0001	-2.42	< 0.0001
Lactate (mmol.L^− 1^)	-0.02	< 0.0001	-0.56	0.002

*VmMCA* mean velocity of middle cerebral arterys; *VsMCA* systolic velocity of middle cerebral arterys; *VdMCA* diastole velocity of middle cerebral arterys; *PaCO*_*2*_ partial pressure (arterial) of carbon dioxide; *PaO*_*2*_ partial pressure (arterial) of oxygen; *SaO*_*2*_ oxygen saturation of blood; *ScO*_*2*_ cerebral oxygen saturation

### Comparison between UF and CE groups after propensity score matching (PSM-UF group and PSM-CE group, respectively)

PSM-UF group (*n* = 55) and PSM-CE group (*n* = 89) were well balanced in age (*P* = 0.09), BSA (*P* = 0.10), CPB time (*P* = 0.23) and STS-EACTS Mortality Category (*P* = 0.36) (see Table 3). The statistical results found in the EEG abnormalities and clinical outcomes between the groups before matching remained similar to those after matching (see Tables 3 and 4). The incidences of the lack of return to normal background by 48 h (*P* = 0.007) and seizures (*P* = 0.009) were significantly less in PSM-UF group. No significant difference was found in the incidence of the lack of return to normal SWC by 48 h (*P =* 0.21) between the two groups (see Table 3). The degree of background abnormalities significantly decreased over 48 h in both groups (*P*_*time*_ < 0.0001), and was significantly less in PSM-UF group compared to PSM-CE group (*P*_*group*_ = 0.02) (see Table 4). Although the number of spike/sharp waves was not decreased over the 48 h in either groups (*P*_*time*_ = 0.98), it was significantly lower in PSM-UF group compared to the PSM-CE group (*P*_*group*_ = 0.008) (see Table 4).

Compared to PSM-CE group, the increase in ScO_2_ over 48 h were significantly greater in PSM-UF group (*P* < 0.0001) (see Tables 3 and 4). MAC velocities (including mMAC, sMAC, dMAC) increased significantly over 48 h in both groups (*Ps* < 0.0001), and was significantly higher in PSM-UF (*Ps* < 0.05) (see Table 4). The level and trend of SaO_2_ were not significantly different between the two groups (*P*_*time*_ = 0.34, *P*_*group*_ = 0.73). PaO_2_ slightly decreased over 48 h in both groups (*P*_*time*_ < 0.0001) but was not significantly different between the two groups (*P*_*group*_ = 0.45). During 48 h, PaCO_2_ in PSM-UF was significantly related to time after polynomial transformation, with an early fast decrease in the first 6 h (parameter estimate_time_ = -0.44, *P*_*time*_ = 0.02) and followed by a gradual increase thereafter (parameter estimate_time_^2^ = 0.009, *P*_time_^2^ = 0.02). The overall level of PaCO_2_ during 48 h was significantly higher in PSM-UF group compared to PSM-CE group (parameter estimate = 7.09, *P* < 0.0001) (see Table 4). The level of lactate decreased significantly in both groups (*P*_*time*_ < 0.0001) but was not significantly different between the two groups (*P*_*group*_ = 0.34) (see Table 4). SBP and DBP decreased in both groups over 48 h (*Ps* < 0.0001), and were not significantly differen*t* between the two group (*P* = 0.42 and *P* = 0.97, respectively). Heart rate increased slightly in both groups (*P* < 0.0001), but was lower in the PSM-UF group (*P* = 0.03). However, the brain injuries detected by MRI before discharge were not statistically different between the two groups (*P*_*group*_ = 0.86). The doses of milrinone, epinephrine and dopamine significantly decreased over 48 h in both groups (*Ps* < 0.0001), and was significantly less in PSM-UF group (*Ps* < 0.0001). In addition, the durations of CICU and hospital stay were significantly shorter in PSM-UF group (*Ps* < 0.0001) (see Table 4).

**Table 3:** Clinical characteristics in PSM-UF and PSM-CE groups after propensity score matching

Variable	PSM-CE (*n* = 89)	PSM-UF (*n* = 55)	*P*-value
Sex, n (%)			0.81
Male	50(56)	32(58)	
Female	39(44)	23(42)	
Age (day)	190.9 ± 131.1	209.2 ± 101.1	0.09
Weight(kg)	6.28 ± 1.70	6.61 ± 1.40	0.23
BSA (m^2^)	0.333 ± 0.06	0.345 ± 0.05	0.10
CPB time (min)	85.09 ± 50.0	80.24 ± 31.3	0.28
STS-EACTS Mortality Category, n (%)			0.36
1	50(56)	33(60)	
2	34(38)	16(29)	
3	1(1)	3(5)	
4	4(4)	3(5)	
EEG abnormalities (*n* = 352)			
Seizures, n (%)	10(11)	0(0)	0.007
Lack of return to normal background by the 48th hour, n (%)	21(24)	4(7)	0.009
Lack of return to normal SWC by the 48th h, n (%)	9(10)	2(4)	0.21
Brain injury degree on MRI (*n* = 80)			0.86
Normal, n (%)	29(43)	7(54)	
Mild, n (%)	35(52)	6(46)	
Severe, n (%)	3(4)	0(0)	
Length of CICU stay (day)	6.0 ± 5.6	2.0 ± 1.4	< 0.0001
Length of hospital stay (day)	13.3 ± 8.5	9.6 ± 4.1	0.002

*BSA* body surface area; *CPB* cardiopulmonary bypass; *STS-EACTS* Society of Thoracic Surgery-European Association of Cardiothoracic Surgery; *CICU* cardiac intensive care unit, *SWC* sleep-wake cycling

**Table 4:** Comparison of the continuous variables of EEG, hemodynamics, inotropes and ScO_2_ during the first 48 h after CPB between PSM-UF and PSM-CE groups after propensity score matching

Variables	Time		Group	
	Parametesr estimate	*P*-value	Parametesr estimate	*P*-value
Degree of EEG background abnormalities	-0.006	< 0.0001	-0.18	0.02
Spikes /sharp waves (times.min^− 1^)	-0.001	0.98	-9.19	0.008
Heart rate (times.min^− 1^)	0.12	< 0.0001	-8.06	0.03
SBP (mmHg)	-0.05	0.0067	1.26	0.42
DBP (mmHg)	-0.10	< 0.0001	0.04	0.97
VmMCA (cm.second^− 1^)	0.62	< 0.0001	6.49	0.02
VsMCA (cm.second^− 1^)	0.80	< 0.0001	8.19	0.05
VdMCA (cm.second^− 1^)	0.45	< 0.0001	4.30	0.05
ScO_2_ (%)	0.31	< 0.0001	7.63	< 0.0001
PaO_2_ (mmHg)	-0.47	< 0.0001	4.99	0.45
SaO_2_ (%)	0.006	0.34	0.23	0.73
PaCO_2_ (mmHg)	-0.44 0.009	0.02 0.02	7.09	< 0.0001
Milrinone (mcg.kg^− 1^.min^− 1^)	-0.003	< 0.0001	-0.17	< 0.0001
Epinephrine (mcg.kg^− 1^.min^− 1^)	-0.0005	< 0.0001	-0.03	< 0.0001
Dopamine (mcg.kg^− 1^.min^− 1^)	-0.04	< 0.0001	-2.24	< 0.0001
Lactate (mmol.L^− 1^)	-0.01	< 0.0001	-0.09	0.34

*VmMCA* mean velocity of middle cerebral arterys; *VsMCA* systolic velocity of middle cerebral arterys; *VdMCA* diastole velocity of middle cerebral arterys; *PaCO*_*2*_ partial pressure (arterial) of carbon dioxide; *PaO*_*2*_ partial pressure (arterial) of oxygen; *SaO*_*2*_ oxygen saturation of blood; *ScO*_*2*_ cerebral oxygen saturation

## Discussion

The present study demonstrated that the UF patients before propensity score matching had better early postoperative outcomes with shorter duration of CICU and hospital stay and lower dosages of inotropic and vasoactive agents as well as lactate level compared with CE patients. Importantly, such statistical significance mostly remained after propensity score matching, i.e. PSM-UF group, confirming the beneficial impact of UF on early postoperative outcomes. These are largely consistent with findings in previous studies [1–3]. For the UF patients, there are several advantages in the postoperative care process, including no procedure for mechanical ventilation weaning, reduced administration of pain management, which facilitate earlier improvement in hemodynamics. These may be the reasons why patients were discharged more quickly than CE group.

More specific in the present study is the finding of the beneficial effect of UF on EEG abnormalities shown both before and after propensity score matching. We comprehensively assessed EEG background (including SWC) and discharge abnormalities (seizures, spikes/sharp waves) in the early postoperative period. All these EEG abnormalities except SWC were significantly less severe in the UF patients compared to the CE patients. We further analyzed the potential underlying mechanisms in terms of systemic and cerebral hemodynamics and oxygenation for the better EEG findings in the UF patients. We found that while heart rate was significantly slower in UF group and PSM-UF group, arterial blood pressures were not significantly different between groups. Further, while SaO_2_ and PaO_2_ were not significantly different especially after matching, PaCO_2_ during the 48 h after CPB in UF group and PSM-UF group was substantially higher by a mean of about 6 mmHg than that in CE group, at least partly attributable to the better cerebral perfusion with significantly higher ScO_2_ and cerebral blood flow velocities.

The cerebral circulation can be regulated by PaCO_2_. In physiological conditions, hypercapnia increases while hypocapnia decreases cerebral blood flow [15, 16]. Carbon dioxide dilates cerebral arteries and increases cerebral blood flow. Furthermore, studies have demonstrated that moderate hypercapnia enhances cerebral blood flow, improves oxygen transport, and decreases oxygen consumption in newborns following the Norwood procedure, a type of cardiac surgery [17]. In our study, PSM-UF group had an initial hypercapnia on arrival in the CICU, whereas PSM-CE group had hypocapnia in the early hours. Therefore conventionally ventilated patients, hypocapnia should be avoided.

In addition to PaCO_2_, the reasons for the higher ScO_2_ and reduced EEG abnormalities in the UF patients may also include the less use of sedative drugs and the patient’s wakefulness. Sedative and anesthetic drugs have an effect in electricity [18]. Although sevoflurane has become increasingly popular in pediatric anesthesia, some have reported seizure-like convulsive movement as effects of sevoflurane anesthesia [19–21]. The anesthesiologist assessed that the UF patients could be withdrawn from mechanical ventilation before leaving the operating room based on the intraoperative assessment (including surgery, circulation, respiration, internal environment during the operation), so sevoflurane was stopped when the sternum was closed in order to make patients awake quickly, although no seizures were found in either of the two groups. However, to date, no evidence has been found to suggest that short-term sevoflurane exposure in children can influence electroencephalographic activity at 48 h postoperatively. Furthermore, the CE patients often need excessively deeper sedation levels (dexmedetomidine, sufentanil and midazolam) in order to avoid discomfort and respiratory resistance in the CICU, which may help to reduce the occurrence of EEG discharge abnormalities.

Furthermore, it has been reported that increased cerebral oxygen consumption in case of increased metabolism is normally met by an increase of cerebral blood flow, the so-called neurovascular coupling [22]. Compared with the UF patients, CE patients were more sedated, so their brain metabolism and oxygen consumption may be lower. On the contrary, the UF patients were in the awaked state, likely leading to increased cerebral oxygen consumption as well as cerebral perfusion, which may be used to explain higher cerebral blood flow velocities in addition to the effect of higher PaCO_2_ compared to CE patients. Sulg and Ingvar et al. have shown a high correlation between the EEG activity and cerebral oxygen consumption, indicating that the increased brain metabolism is clearly reflected in EEG [23, 24]. This might be at least partially attributable to the improved EEG, particularly EEG background, in the UF patients. To date, no definitive evidence has been established to confirm that hypercapnia affects EEG activity by altering cerebral perfusion. Taken together, the UF patients was in a state of minimal medical intervention, with potentially better adjustment of internal environment, resulting in quicker and better recovery in brain electrical activity. The brain injuries detected by MRI were not statistically different between the two groups. This finding suggests that abnormalities in electroencephalography (EEG) may be confined to the perioperative period (within 48 h), or that such EEG differences have not yet resulted in long-term changes in imaging. However, we are still following up on whether there is a difference in EEG after discharge between the two groups of patients.

### Limitations

This study has a couple of limitations: (1) This is a post hoc analysis of previously collected data using propensity score-match method, rather than the gold standard randomized and control study. Nonetheless, the information obtained from the present study remains valid since propensity score-match method is a valid method to compare between control and intervention groups and has been used in previous studies [3]. (2) The intermittent intravenous infusion of midazolam was administered by the bed-side nurse when necessary, but the frequency and doses were often not recorded, so we could not quantitatively compare the differences in sedation between two groups. (3) We studied the early postoperative brain injury on EEG and clinical outcomes. Further studies are warranted to examine the effects of UF on long-term neurodevelopmental outcomes, which is expectedly improved given our recent finding in the association of early postoperative EEG abnormalities and adverse neurodevelopmental outcomes [25]. (4) Propensity score matching was based on available baseline and surgical variables but could not account for real-time intraoperative clinical judgments regarding arterial blood gases, lung compliance and ventilator support that determined suitability for UF extubation. Therefore, despite statistical matching, residual confounding due to unmeasured perioperative factors may persist.

## Conclusions

In this propensity score-matched analysis, ultra-fast-track extubation was associated with a reduced incidence of specific postoperative EEG abnormalities (spikes/sharp waves and background abnormalities) and was also associated with improved early postoperative outcomes, including reduced vasoactive-inotropic requirements, and shorter duration of CICU and hospital stay in children with CHD undergoing CPB.

## Data Availability

The datasets generated during and/or analysed during the current study are available from the corresponding author on reasonable request, subject to the required ethical approval for data sharing.

## Abbreviations

UF: Ultra-fast-track extubation
CE: Intensive care unit extubation
BSA: Body surface area
CHD: Congenital heart disease
CPB: Cardiopulmonary bypass
EEG: Electroencephalographic
MRI: Magnetic resonance imaging
SWC: Sleep-wake cycling
CICU: Cardiac intensive care unit
STS-EACTS: Society of thoracic surgery-european association of cardiothoracic surgery
